# Postpartum Abdominopelvic Stabilization Dysfunction and Bowel Function: A Proposed Mechanistic Model Linking Pressure Control, Visceral Mechanics, Transit, and the Intestinal Environment—A Critical Narrative Review

**DOI:** 10.3390/nu18213704

**Published:** 2026-11-08

**Authors:** Agnieszka I. Mazur-Bialy

**Affiliations:** Department of Biomechanics and Kinesiology, Institute of Physiotherapy, Faculty of Health Sciences, Jagiellonian University Medical College, Skawińska 8, 31-066 Krakow, Poland; agnieszka.mazur@uj.edu.pl; Tel.: +48-12-421-93-51

**Keywords:** postpartum recovery, abdominopelvic stabilization, diaphragm, pelvic floor, visceral displacement, intestinal motility, colonic transit, gut microbiota

## Abstract

Postpartum bowel symptoms are often attributed to tissue injury, pelvic-floor trauma, or abdominal diastasis. Structural measures do not capture task-specific diaphragm–abdominal-wall–pelvic-floor function. This critical narrative review proposes an abdominopelvic-to-gut model for a susceptible postpartum subgroup. PubMed/MEDLINE, Scopus, and Web of Science were searched till 8 August 2026; 40 reports were included (12 Core; 28 Supporting). All 34 primary reports among the 40 retained received preliminary design-specific appraisal. Route A connects pressure–outlet dysfunction with inefficient evacuation, retention, and possible transit delay. Its relatively closer support comes from non-postpartum physiological studies and selected postpartum anorectal and symptom outcomes, without demonstrating stabilization-mediated transit change after childbirth. Route B links respiratory mechanics to bowel displacement, accommodation, and possible pressure-dependent fluid transport; its postpartum bowel effects remain hypothetical. Transit and stool residence may connect mechanics with microbial metabolism, while diet and lactation can modify or independently generate intestinal changes. The contribution is an integrated, falsifiable framework, not validation of the full causal chain. Functional, bowel, and nutritional measurements must be synchronized to test its links.

## 1. Introduction—Postpartum Recovery and the Abdominopelvic-to-Gut Model

The six-week puerperium does not capture full functional recovery [1,2]; medium- and long-term consequences remain underrecognized [3]. Among 1117 women assessed 3–6 months after childbirth, only 42.5% met all recovery criteria. Median time to full recovery was 15 weeks [4]. We use postpartum recovery for the broader first year after birth and puerperium for the early six-week interval. Symptoms remain common beyond that interval. A prospective cohort of 208 women recorded a decrease in any pelvic-floor symptom from 92.8% during pregnancy to 73.6% at one year. Constipation decreased from 65.3% to 42.8%, and urinary incontinence from 56.8% to 35.1% [5]. Among 408 women assessed 3–12 months postpartum, constipation affected 40.7%, flatulence 34.1%, urinary incontinence 25.2%, and fecal incontinence 2.9% [6]. These cohorts document persistent co-occurring symptoms but cannot resolve a common mechanism.

Recovery occurs alongside endocrine withdrawal, tissue repair, uterine involution, and lactation, as well as time-dependent immune changes [7,8]. Pelvic-floor dysfunction spans closure and evacuation: reduced support, force, or reflex activation can impair continence. Insufficient relaxation or mistimed activation can instead increase outlet resistance [9,10]; these patterns can coexist, but symptom clustering cannot identify the dominant driver. Possible drivers include injury, altered neuromuscular control, pain, bowel behavior, and other postpartum exposures.

Current postpartum explanations usually emphasize obstetric injury, sphincter or levator defects, and inter-recti distance. However, a systematic review found no eligible prospective evidence linking obstetric events to postpartum constipation with standardized validated measures [11]. Immediate postpartum ultrasound also failed to predict constipation at follow-up [12]. Structural findings cannot describe interactions among the diaphragm, abdominal wall, pelvic floor/outlet, and intra-abdominal pressure during breathing, loading, or evacuation. The missing level is task-specific abdominopelvic stabilization.

Here, abdominopelvic stabilization denotes the task-dependent capacity of the diaphragm, abdominal wall, and pelvic floor to generate and transmit pressure while controlling trunk support, visceral displacement, and the appropriate outlet response. We use stabilization dysfunction for an altered response during a specified breathing, loading, or evacuation task, rather than for diastasis recti abdominis (DRA), weakness, incontinence, or poor coordination alone. Its proposed operational assessment combines task-specific pressure, abdominal-wall behavior, diaphragm motion, and pelvic-floor or anal activation/relaxation. This is an author-defined construct without validated diagnostic thresholds [9].

Candidate subgroups include women with impaired support or closure, women with excessive outlet resistance or inadequate relaxation during evacuation, and women with altered thoracoabdominal accommodation and disproportionate distension; mixed presentations are possible. These are hypotheses for stratification, not validated postpartum classes. Injury, DRA, delivery mode, pain, pre-existing bowel symptoms, and lactation should be recorded separately rather than used to assign a functional subgroup.

For a susceptible subgroup, postpartum abdominopelvic stabilization dysfunction may alter bowel function through two proposed mechanical pathways with unequal evidentiary support. The relatively better-supported pressure–outlet pathway (Route A) connects impaired pressure generation or transmission and an inadequate pelvic-floor/anorectal response with inefficient evacuation and retention. The parallel respiratory-mechanics pathway (Route B) concerns altered diaphragm–abdominal-wall mechanics, respiratory bowel displacement, abdominal accommodation, and pressure-dependent fluid movement. Greater diaphragmatic excursion is not assumed to accelerate transit, because respiratory displacement and intrinsic peristaltic motion are distinct phenomena. Convergence on stool residence, transit, and luminal conditions remains to be established postpartum. Diet, lactation, medication, pain, activity, adiposity, and delivery-related exposures may modify these links or produce intestinal changes independently. We call this the abdominopelvic-to-gut model and evaluate each connector against direct postpartum evidence, indirect mechanistic support, and measurements capable of confirming or falsifying the proposed sequence. This distinction is particularly relevant for nutritional and microbiome research, because stool residence, transit, and contemporaneous dietary substrate exposure can independently shape microbial composition and metabolite readouts.

For nutritional interpretation, postpartum studies already document changing dietary intake and maternal microbial trajectories but generally do not measure transit or stabilization [13,14,15,16,17]. They therefore provide postpartum context, not direct evidence for a postpartum transit–microbiota mechanism. The gap across existing bowel, pelvic-floor, recovery, and maternal-microbiota reviews is the absence of a common task-based framework that distinguishes tested connectors from transfer assumptions [11,13,18]. Our objectives are to identify postpartum functional and bowel findings, assess the applicability and methodological limitations of mechanistic evidence, and specify observations that would support or contradict each proposed connector. The novelty is integration and explicit testing of established mechanisms in a postpartum setting, not discovery of a new physiological mechanism.

## 2. Materials and Methods

This critical narrative review was informed by a structured search of PubMed/MEDLINE, Scopus, and Web of Science finished till 8 August 2026 (June–August 2026). The documented strategy combines a postpartum block (postpartum, postnatal, puerperium, after childbirth) with a functional block (diaphragm, abdominal wall/muscles, pelvic floor, abdominopelvic/lumbopelvic function, core stabilization, intra-abdominal pressure) and a bowel block (motility, transit, peristalsis, constipation, defecation/outlet dysfunction, bloating/distension, gut–brain interaction disorders). The database-specific working strategies and the recorded additional gap-check searches are provided in Supplementary Table S1.

The databases yielded 382 records (PubMed/MEDLINE 91, Scopus 187, Web of Science 104). After removal of 173 duplicates, 209 records were screened and 152 excluded at title/abstract level. Fifty-seven reports were sought; 10 could not be retrieved. Of 47 full texts assessed for eligibility, seven were excluded, leaving 40 reports included (12 Core; 28 Supporting). Counts refer to reports rather than necessarily unique studies; related Motherhood and Pelvic Health (MAP) cohort publications were flagged as one report family. Supplementary Figure S1 shows the adapted flow diagram. The database exports and 57-report screening/extraction ledger were retained; all 57 sought reports were matched to the exports by DOI, PMID or title. No publication-year, language or study-design restrictions were applied to the searches. All 40 included reports were in English. The literature search, title and abstract screening, full-text eligibility assessment, data extraction, and appraisal using the Joanna Briggs Institute (JBI) checklist were performed by a single author. Uncertainties were addressed by repeated examination of the original publications.

The 40 included reports were selected for relevance to postpartum abdominopelvic or pelvic-floor function and bowel symptoms, with reviews and consensus retained for clinical or mechanistic context. Intervention, cohort, cross-sectional, prevalence, and case–control designs were represented. Core denotes narrative priority for a proximal connector or an interpretation-changing finding; supporting denotes contextual or overlapping evidence charted without repetition. This classification is not a quality ranking: the 12 Core reports include seven primary reports and five secondary sources. Extraction fields in Supplementary Table S2 include design, population and timing, exposure, bowel findings, directness, limitations, and family reports. Structural, continence and modifier reports without direct bowel physiology are included as contextual components rather than pathway tests.

Evidence was synthesized by connector rather than pooled. Supplementary Table S2 charts all 40 included reports; Supplementary Table S3 provides preliminary item-level appraisal for all 34 primary reports in that set (35 matrices because Palmieri has complementary assessments). Obstetric pelvic-floor terms follow the ICS/IUGA consensus; “abdominopelvic stabilization dysfunction” is used here as an author-defined mechanistic construct, not a diagnostic label [9].

A preliminary methodological appraisal was prepared for all 34 primary reports using fixed 2017 JBI design-specific checklists [19]. Item-level decisions were recorded as yes, no, unclear, or not applicable, with source locators; no summed quality score or GRADE rating was assigned. The 34 report-level appraisals have 35 item-level matrices because Palmieri was assessed for complementary prevalence and cross-sectional questions (Supplementary Table S3).

## 3. Results of the Evidence Synthesis: Stabilization Mechanics

### 3.1. Diaphragm–Abdominal Wall–Pelvic Floor Mechanics

Diaphragmatic contraction during inspiration displaces the diaphragm caudally and raises intra-abdominal pressure (IAP). For a given descent, the pressure rise depends on abdominal-wall compliance: a more compliant wall accommodates displacement with a smaller IAP increase [20]. Dynamic MRI in healthy women shows synchronous caudal movement of the diaphragm and pelvic floor during inspiration and cranial return during expiration [21]. The cited compliance illustration is a postsurgical case report, not a postpartum comparison, and the MRI study involved eight healthy nulliparous women [20,21]. Neither establishes postpartum bowel effects.

Pressure strategy changes with the task. During defecation, diaphragm and abdominal-wall contraction increase abdominal and rectal pressure while the puborectalis and anal sphincters relax, creating a rectum-to-anal pressure gradient [22,23]. MRI–manometry showed that successful evacuation combines abdominal expansion, anorectal descent, rectal pressurization, anal opening, and emptying; failure was associated with smaller changes in each component [24]. Ultrasound–manometry likewise identified aberrant abdominal recruitment and abnormal abdomino-anal pressure coupling in defecatory disorders [25]. Across clinical stabilization tests, IAP also covaried with abdominal-wall tension, reinforcing the need to measure function rather than infer pressure control from morphology [26].

Postpartum measurements show task-dependent, nonuniform differences. In a cross-sectional study of 162 women (54 with postpartum stress urinary incontinence (SUI), 54 continent postpartum women and 54 nulliparous controls) the SUI group showed less transversus abdominis thickening and greater caudal bladder-base displacement during selected respiratory tasks. During maximum deep respiration and cough, diaphragm excursion was smaller in both postpartum groups than in nulliparous controls, without a significant difference between the two postpartum groups [27]. Among 90 women within six months of vaginal birth, drawing-in, head lift, and active hip/knee flexion produced pelvic-organ descent or hiatal enlargement. Simultaneous pelvic-floor contraction reduced but did not always prevent these changes [28]. Longitudinal ultrasound also showed increased perineal-body and anorectal-junction mobility after childbirth (both *p* < 0.001), related to levator-hiatus distensibility [29]. Reviews across respiratory and physical-loading contexts describe similarly variable pelvic-floor responses [30,31]. These studies support multicomponent functional assessment; their outcomes concern respiratory coupling, morphology, and support, not standardized IAP, bowel evacuation, or transit. Ultrasound excursion and thickening are not direct measurements of force, pressure transmission, or intestinal function.

Task specificity explains why continence and evacuation can diverge: reduced force, structural or neural injury, or inadequate reflex activation may compromise urinary or anal closure. Insufficient relaxation or mistimed activation may instead increase outlet resistance; both patterns can coexist [9,23]. At a mean 14 months after vaginal birth, fecal urgency affected 11.7% and weekly flatus leakage 8.7% of 207 women. Among those examined, 9.3% had an unrecognized third-degree sphincter injury [32]. A national obstetric and sphincter injury (OASI) cohort found anal incontinence in about one third at 12 months, irrespective of body mass index; urinary incontinence increased with body mass index [33]. These findings document heterogeneous closure dysfunction; continence outcomes may inform the stabilization phenotype but cannot serve as surrogates for evacuation or transit.

Inter-recti distance (IRD) measures morphology, not stabilization. Among 224 symptomatic postpartum women, crude associations between IRD and demanding activities disappeared after adjustment for body mass index and parity [34]. Repeated assessments in a prospective cohort found no poorer pelvic-floor function or higher urinary-incontinence/prolapse likelihood with DRA, but follow-up loss and palpation-based DRA classification limit precision; non-significance is not proof of equivalence [35]. In the MAP cohort, IAP during lifting and trunk endurance at 5–10 weeks did not predict one-year support or symptom burden [36]. At one-year, general muscular fitness did not consistently modify the IAP–support relation [37]. These studies concern morphology or pelvic support, not evacuation or transit. Functional assessment must capture diaphragm excursion, abdominal-wall compliance and tension, IAP generation, and outlet response. The related MAP publications sample one cohort and do not provide independent replication.

### 3.2. Respiratory Bowel Displacement and Abdominal Accommodation

Respiration produces substantial diaphragmatic and upper-abdominal organ displacement [38,39], and a respiratory component is measurable in the bowel. Dynamic MRI in 20 healthy adults showed that respiratory-motion correction reduced region-propagation error by 90% in the small bowel and 55% in the colon before motility quantification [40]. This is a technical improvement in separating motion components, not evidence that breathing increased peristalsis. Respiratory bowel displacement is therefore measurable and physically distinct from intrinsic contractile motility. The sonographic sliding sign used at repeat caesarean may indicate adhesions [41], but it was developed for a different question and cannot quantify physiological respiratory bowel motion.

Gut volume also elicits a diaphragm–abdominal-wall response: during experimental colonic gas loading, healthy participants relaxed the diaphragm and increased anterior abdominal-wall tone. Patients with bloating showed paradoxical diaphragmatic contraction and internal-oblique relaxation during spontaneous distension [42]. A separate paired-CT study during spontaneous distension in 47 functional patients showed only a small content increase (0.3 ± 0.1 L), with diaphragm descent (12 ± 3 mm) and anterior-wall protrusion (14 ± 2 mm), consistent with caudo-ventral redistribution. Its nine-patient dysmotility comparison showed that distension can instead involve genuine content accumulation [43]. A later placebo-controlled trial reported correction of the abnormal thoracoabdominal pattern and a 66% reduction in distension symptoms [44]. These non-postpartum studies measure accommodation; they do not show that correcting it improves transit. The final full methods of the biofeedback trial remain unavailable for complete appraisal. Accommodation is therefore a plausible Route B intermediate, but its postpartum relevance and relation to intrinsic motility and colonic transit requires independent recording.

### 3.3. Respiratory Pressure Gradients and Fluid Transport

Respiration changes pressure gradients in venous, lymphatic, and peritoneal compartments. Respiration also alters venous dynamics. In trained abdominal breathers, inferior vena cava (IVC) collapsibility increased during slow respiration with an inspiratory pause compared with normal respiration (62 ± 19% vs. 48 ± 19%) and was greater than in controls during normal respiration (48 ± 19% vs. 26 ± 12%) [45]. Terminal thoracic-duct diameter increased modestly during inspiration [46], while peritoneal liquid drainage varied with transdiaphragmatic pressure and respiratory frequency in spontaneously breathing rabbits [47]. Human diameter/collapsibility measurements are morphological proxies, not measurements of volumetric venous or lymphatic flow; the rabbit experiment measured a distinct peritoneal transport process. These findings motivate separate tests of pressure-sensitive fluid transport. Its postpartum magnitude and relation to intestinal perfusion, local tissue-fluid exchange, intrinsic motility, or transit remain unknown. The available data do not support a generalized ‘drainage’ claim.

## 4. Results of the Evidence Synthesis: From Abdominopelvic Stabilization Dysfunction to Bowel Function: The Evacuation–Transit Route

Route A is the better-supported proximal pathway: impaired pressure generation or transmission combined with inadequate pelvic-floor/anorectal relaxation can reduce evacuation efficiency, promote retention, and in some patients prolong transit. Route B addresses bowel displacement and abdominal accommodation separately. Better-supported means that more proximal connectors have direct human physiological tests and selected postpartum anorectal observations. It is a qualitative judgment of directness and relevance, not a pooled effect, quality score, or demonstration of the complete postpartum chain.

### 4.1. Postpartum Evidence: Function Is Not Explained by Structure Alone

Anorectal symptoms can persist after childbirth [18,48], but obstetric anatomy explains postpartum constipation poorly. A 2021 systematic review identified no eligible prospective study linking obstetric events to postpartum constipation with standardized validated measures [11]. At three months, complete puborectalis avulsion was associated with lower passive stiffness, strength, contraction speed, and endurance. Bowel-pattern, bowel-control, and colorectal-impact scores did not differ, although chronic constipation was excluded [49]. Among 182 women, constipation affected 43%; immediate postpartum ultrasound did not predict it, whereas constipation at term did (aOR 3.73, 95% CI 1.81–7.69) [12]. Another cohort identified functional constipation in 123/377 women at six weeks, with an association with vaginal wind (OR 2.41, 95% CI 1.43–4.07). Delivery mode and pelvic-floor strength were not independent predictors of vaginal wind [50]. Vaginal wind is not an evacuation or transit endpoint. Among 845 women assessed at one-month, gestational constipation was associated with abnormal resting pelvic-floor surface electromyography (sEMG) (OR 1.553, 95% CI 1.022–2.359) [51]. Evacuation physiology was not measured, so the direction of this association remains uncertain.

Postpartum manometry and randomized trials sample different layers of the proposed phenotype. Among 127 women evaluated for postpartum constipation, manometry showed delivery-mode-related differences in maximal contracting sphincter pressure but did not assess abdominal mechanics, dyssynergia, or transit [52]. A 2024 trial assigned 56 women with constipation after caesarean delivery to rehabilitation with or without transcutaneous tibial nerve stimulation (TTNS). Both groups received diaphragmatic breathing, abdominal hollowing/bracing, and behavioral advice. Symptoms, quality of life, and stool form improved in both groups; final Patient Assessment of Constipation Symptoms (PAC-SYM) and Patient Assessment of Constipation Quality of Life (PAC-QOL) favored TTNS, but the common rehabilitation contribution remained inseparable [53]. A 2026 trial assigned 40 primiparous women with pelvic floor dyssynergic defecation to TTNS or sham alongside standard rehabilitation. Both groups improved; response was reported in 18/20 versus 13/20 women (90% versus 65%). These counts do not support the article’s claimed between-group significance (two-sided Fisher exact *p* = 0.127, recalculated). Selected pelvic-floor sEMG and manometric values were also reported, but measurement and statistical-reporting inconsistencies limit interpretation [54]. Neither trial establishes normalized evacuation mechanics, measured transit, or mediation through stabilization.

A third multicenter trial compared a holistic programme—abdominal breathing, pelvic and core activation, bridging, and progressive stability exercises—with Kegel training. Both approaches were combined with electrical stimulation and biofeedback. In total, 120 women were allocated and 87 analyzed (41 and 46), despite inconsistent enrollment wording in the article. At three months, type I pelvic-floor muscle strength and urinary-leakage frequency favored the postural programme. Total Pelvic Floor Distress Inventory-20 (PFDI-20) and its colorectal–anal distress inventory-8 subscale (CRADI-8) did not differ between groups [55]. Reported benefits were confined to selected pelvic-floor and continence outcomes; attrition and inconsistent denominators/statistical entries reduce confidence. A small trial also added whole-body vibration to pelvic-floor and static abdominal exercise plus diet. An incremental symptom signal was reported, but shared co-interventions preclude attribution to stabilization alone; the small trial and inconsistent statistical reporting limit confidence in the added whole-body vibration (WBV) effect [56].

### 4.2. Pressure–Outlet Coordination: Human Mechanistic Evidence and Postpartum Transfer

Defecation directly tests how pressure control affects a gastrointestinal task. Normal evacuation requires coordinated colonic/anorectal, pelvic-floor, and neural activity [23,57]. In non-postpartum women, simultaneous ultrasound–manometry and MRI–manometry link failed evacuation to abnormal abdominal recruitment and inadequate rectal pressurization. Insufficient anal relaxation and reduced anorectal or abdominopelvic motion are also present [24,25]. These findings define the proximal Route A mechanism without implying that every bowel effect of stabilization dysfunction operates through defecation.

A one-year randomized biofeedback trial provides causal evidence for this local link: defecatory physiology and bowel frequency improved, and colonic transit normalized only in the biofeedback group [58]. The study was not postpartum, but it shows that correcting pressure–outlet dysfunction can change transit when outlet delay contributes to retention. The small, selected long-term follow-up limits precision and generalizability; this local causal evidence cannot establish a postpartum respiratory–intestinal pathway.

### 4.3. Transit, Motility, and the Bridge to the Intestinal Environment

Respiratory bowel displacement, intrinsic motility, evacuation, and colonic transit are distinct measurements. Dynamic MRI can remove respiratory motion before quantifying small-bowel and colonic motility [40]. Thoracoabdominal biofeedback can also correct an abnormal diaphragm–abdominal-wall pattern without demonstrating a transit effect [44]. Route A links outlet dysfunction to retention. A direct Route B test should pair respiratory mechanics with bowel displacement or abdominal accommodation. An independent motility or transit measure is needed to avoid inferring propulsion from visible movement.

Abdominal massage provides an indirect mechanics–transit signal. A 2025 meta-analysis of 23 studies found a 21.5 h shorter gut transit time versus control [59]. Only three studies contributed transit data, and heterogeneity was substantial (*I*^2^ = 65%). The intervention does not isolate diaphragm, IAP, or pelvic-floor mechanisms and is not postpartum evidence.

Transit matters because stool consistency and colonic residence are associated with microbial composition and metabolism [60,61]. Longer transit can accompany higher richness and increased protein-derived metabolites; greater diversity is not necessarily a health benefit [61]. Yu et al. combined radiopaque-marker transit, high-resolution anorectal manometry, fecal 16S profiling, and fecal short-chain fatty acids (SCFAs) [62]. In women with chronic constipation, mucosal and fecal microbial features were also related to transit and methane production [63]. A separate stratified study found microbial differences across constipation phenotypes but remained cross-sectional [64]. Reported associations between selected evacuation-test abnormalities and microbial overgrowth were test- and threshold-dependent, with no association between the balloon expulsion test (BET) and aspirate-defined small-intestinal bacterial overgrowth (SIBO). Referral selection, unadjusted analyses and numerical reporting inconsistencies limit interpretation; temporal direction remains unestablished [65]. Collectively, these studies connect stool residence and outlet phenotypes with the intestinal environment, but they are cross-sectional or non-postpartum and do not identify respiratory mechanics as the initiating exposure.

Two multimodal DRA cohorts reported bowel-symptom changes after intervention. After diastasis repair, 80 symptomatic women reported overall GI improvement in 97.5%; bloating fell from 95% to about 10%, and constipation from 50% to 15% after treatment, but its baseline percentage differs between the abstract and results [66]. The TOR cohort included 71 women more than one year postpartum with training-resistant DRA and core instability. Preoperative training targeted pelvic-floor activation, uncompensated abdominal-wall tension, and diaphragmatic control. Tailored linea alba repair was followed by four months of progressive rehabilitation. At one year, reported constipation decreased from 21.1% to 1.4% and urinary incontinence from 9.9% to 0%. Self-reported core function also improved markedly [67]. These co-occurring changes are hypothesis-generating. Without controls, they cannot separate surgery, training, rehabilitation, selection, expectation, regression to the mean, or time-related recovery. Neither study measured IAP, evacuation mechanics, or transit, and neither demonstrates that restored stabilization caused bowel improvement.

Route A can be tested as pressure–outlet dysfunction → inefficient evacuation or retention → possible transit prolongation. For Route B, human studies document respiratory bowel displacement and abnormal abdominal accommodation outside the postpartum setting. The unresolved connector is the effect of altered postpartum respiratory mechanics on intrinsic motility or transit. Table 1 summarizes the evidence.

## 5. Results of the Evidence Synthesis: Postpartum Modifiers of Transit and Intestinal Readouts

Section 4.3 places evacuation, retention, stool form, and measured transit between stabilization and intestinal readouts. The present section addresses exposures that can modify those links or generate intestinal change independently. In the literature identified for the present review, no postpartum cohort simultaneously measured stabilization, evacuation or transit, and intestinal readouts longitudinally.

Postpartum diet changes over time: the ECLIPSES cohort recorded declines in fiber and vitamins E and C from pregnancy to postpartum [14]. ORIGINS showed persistently low diet quality (Australian Recommended Food Score, ARFS: 34.0 to 34.8 of 73 points) despite food-group and nutrient shifts by one year postpartum [15]. Fruit, dairy, carbohydrate, saturated-fat, iodine, and folate intake fell, whereas vegetable, meat, and protein intake rose. These changes can alter stool bulk and microbial substrate delivery independently of abdominopelvic function, so diet requires contemporaneous measurement rather than a single baseline covariate.

Maternal microbiome evidence remains limited: in 20 lactating women sampled from day 2 to month 6, community structure remained relatively stable, although several taxa varied with reported nutrient intake [16]. At six weeks, a cross-sectional analysis of 24 dyads compared exclusive with partial human-milk feeding; all mothers were lactating. The maternal Shannon group contrast was not significant (*p* = 0.06), whereas the separately reported univariate-model association was attenuated after BMI-category adjustment. The maternal Sørensen association also did not retain significance in the BMI-inclusive model; Bray–Curtis did not differ [17]. Diet, lactation, and adiposity should therefore be prespecified in postpartum analyses, although small samples prevent reliable independent effect estimates.

Postpartum microbiome trajectories are entangled with adiposity, gestational-diabetes history, delivery-related exposures, cortisol, parity, and microbial metabolic phenotype [73,74,75,76,77,78]. These observational associations make weight trajectory, metabolic status, delivery mode, and antibiotic exposure relevant confounders or interaction candidates. They do not identify pressure control or prove that microbial changes follow a mechanical deficit.

Parallel postpartum cohorts show that common exposures are poor surrogates for stabilization. Early MAP accelerometry had mainly null or nonlinear associations with one-year pelvic-floor outcomes [79]. A related report found no association of pre-pregnancy BMI, gestational gain, or postpartum weight retention with most outcomes, including constipation and anal incontinence [80]. Epidural analgesia was associated with dyspareunia, but not other pelvic-floor symptoms or measured function [81]. These variables remain covariates or interaction candidates; they do not identify task-specific pressure control.

Interventions show that the maternal microbiome can change without correcting mechanics. Perinatal probiotic supplementation increased detection of administered strains in maternal stool [82]. Balanced energy–protein supplements changed microbial diversity, taxa, and microbial functional profiles/pathways [83], while live-culture fermented grain increased alpha-diversity and altered nutritional and inflammatory signatures [84]. Microbial change alone does not establish clinical benefit. None measured abdominopelvic function, evacuation, or transit, making rehabilitation-related microbial change uninterpretable without concurrent diet, lactation, medication, weight trajectory, metabolic status, and stool phenotype.

Stool form and measured transit are candidate mediators; other postpartum exposures are confounders, modifiers, or alternative explanations. An association between pelvic-floor function and vaginal microbiota at 6–8 weeks does not support the gut pathway because the sampled compartment was vaginal [85]. Route B requires a documented mechanical intermediate before intestinal associations are interpreted: bowel displacement, abdominal accommodation, or a prespecified pressure-dependent fluid parameter. A diaphragm–microbiota correlation alone bypasses the proposed sequence. Table 2 summarizes the modifier evidence; Section 7 specifies temporal and mediation tests.

## 6. Results of the Evidence Synthesis: Microbial Metabolites, Barrier Signals, and Potential Feedback

A rigorous test of the full chain would phenotype stabilization and bowel function first, then establish whether changes in evacuation or transit precede intestinal and host-signal changes. The key evidentiary gap is a postpartum cohort measuring abdominopelvic function, evacuation or transit, microbiota/metabolites, and host responses repeatedly within the same temporal sequence [13].

SCFAs are the most developed candidate signals because microbial fermentation links them to epithelial, metabolic, and immune processes [68,69]. In the multilevel constipation cohort of Yu et al., fecal SCFA patterns differed between slow-transit and dyssynergic-defecation phenotypes [62]. Fecal concentration, however, also reflects substrate supply, cross-feeding, epithelial absorption, stool water, and transit; it is not a direct measure of production or systemic exposure. Bile-acid and tryptophan-derived metabolites provide additional pathways [86,87], but their relevance to postpartum functional recovery is unknown.

Barrier markers are further downstream and less specific. Intestinal permeability is only one component of barrier function [88]. Sugar-recovery tests are influenced by several pre-analytical and physiological factors, whereas lipopolysaccharide-binding protein and intestinal fatty acid-binding protein reflect distinct biological processes [70]. Commercial zonulin assays have specificity problems [89,90], and very low circulating lipopolysaccharide concentrations are difficult to quantify reliably [91]. Barrier status should therefore be represented by a convergent panel interpreted alongside sampling time, transit, and clinical context.

Systemic inflammation is equally non-specific. Postpartum cytokine trajectories vary with time and parity [8], while perineal injury can independently increase inflammatory markers [92]. A 2026 cross-sectional surgical study outside the postpartum setting reported higher plasma lipopolysaccharide-binding protein and uterosacral-ligament inflammatory-cell infiltration in unadjusted pelvic organ prolapse (POP)–control comparisons. Microbial richness correlated with prolapse stage before, but not after, age adjustment, whereas selected stage-associated taxa remained significant. Comparable LPS and zonulin values, incomplete confounder control and the cross-sectional design do not establish barrier failure or temporal direction [93]. Immune regulation of muscle repair is established, and animal models support microbiota–muscle signaling [71,72]. The missing experiment is a temporally resolved postpartum study linking an intestinal change to subsequent pelvic-floor, abdominal-wall, fascial, or neural recovery. Table 3 outlines the limitations in interpreting these signals.

## 7. Discussion: Integrated Model Evidentiary Limits

The evidence hierarchy is connector specific. Route A has the closer mechanistic foundation: non-postpartum pressure–outlet studies link measured dysfunction to evacuation, and selected postpartum manometric and rehabilitation reports provide proximal observations. This is not proof that postpartum stabilization deficits initiate transit delay. Route B has measured human displacement and accommodation components, morphological fluid proxies, and a separate animal drainage experiment; their integration into a postpartum intestinal pathway is hypothetical. Transit–microbiota associations are mostly indirect, whereas postpartum nutritional studies establish competing substrate exposures. Barrier, inflammatory, and recovery feedback links are further hypotheses. Methodological appraisal of the 40-report structured-search set identified selected samples, attrition, incomplete confounder control, bundled interventions, self-reported outcomes, and reporting inconsistencies. All 34 included primary reports have preliminary item-level appraisal. Separately cited mechanistic and nutritional studies are not included in this restricted appraisal table. The single-reviewer approach to screening, data extraction, and methodological appraisal represents an additional methodological limitation. Directness labels cannot substitute for these limitations.

The model integrates established pressure–outlet and abdominophrenic concepts with postpartum functional assessment and nutritional interpretation; it does not establish a new physiological mechanism. Contrary observations are informative: DRA width does not consistently track pelvic-floor function, and the postural trial did not improve CRADI-8 relative to its comparator [35,55]. Constipation, straining, pain, reduced activity, and medication can alter motor behavior before a measured stabilization deficit. Diet or lactation can change microbial readouts without any mechanical intermediate. Accordingly, the proposed initiating direction must compete with reverse causality and common-cause explanations; symptom improvement alone does not establish mediation.

The model is a sequence of testable links, not a single axis. Route A connects impaired rectal pressurization or outlet relaxation with inefficient evacuation [24,25]; correction of verified dyssynergia can normalize transit when outlet delay contributes [58]. Route B connects respiratory mechanics with bowel displacement and abdominal accommodation [40,44] and, separately, respiratory changes in thoracic-duct diameter peritoneal drainage, which must not be treated as equivalent flow outcomes [46,47]. Microbial composition and metabolites lie further downstream of stool phenotype and transit [61,62].

### 7.1. Connector-Specific Predictions

The first Route A test is whether task-specific abdominal-wall recruitment, rectal pressurization, and pelvic-floor or anal relaxation explain evacuation better than morphology [24,25]. Postpartum studies should compare these measures with puborectalis avulsion and early ultrasound, which correspond poorly with bowel symptoms [12,49]. A mechanism-focused trial should document improved pressure–outlet mechanics and evacuation before attributing a later transit change to Route A. Transit can normalize when verified outlet dysfunction contributes to delay [58]. Postpartum TTNS data include symptoms, pelvic-floor sEMG, and reported manometric values, but do not establish normalized evacuation, measured transit, or a stabilization-mediated effect; reporting inconsistencies require caution [54]. Respiratory, loading, and defecatory tasks require separate protocols.

For the intestinal arm, longitudinal analyses should test whether retention, stool form, or measured transit mediates associations between stabilization dysfunction and intestinal readouts [61,62]. Models must account for diet, lactation, medication, and other time-varying exposures. Microbial changes that preceded the mechanical or bowel phenotype would oppose the proposed sequence. Changes independent of a verified mechanical/transit intermediate would weaken that proposed mediated link, while remaining compatible with independent nutritional or other pathways.

Evaluation of the parallel respiratory-mechanics pathway begins with direct measurement of bowel displacement or abnormal abdominal accommodation [40,44]. If the fluid-transport arm is retained, investigators should also prespecify a pressure-dependent fluid parameter [46,47]. A claimed transport or perfusion effect requires an actual flow/perfusion measure rather than diameter alone. The measured intermediate can then be related to bowel symptoms, intrinsic motility, transit, or intestinal readouts. A displacement change without a motility effect would validate the mechanical component while narrowing the downstream model.

### 7.2. Falsification and Model Boundaries

The pressure–outlet pathway would lose support if functional measures were unrelated to evacuation or transit after confounder control. Equivalent performance by IRD or failure of a verified correction to improve the corresponding bowel phenotype would also weaken it. Its intestinal extension requires microbial associations to depend on transit or stool phenotype and follow the expected temporal order after dietary adjustment. For Route B, failure to reproduce the proposed displacement, accommodation, or fluid intermediate would close that link. The same applies if the verified intermediate shows no bowel association.

The model need not apply to every postpartum woman. Pain, medication, diet, hydration, lactation, activity, pre-existing bowel disease, metabolic factors, and reverse effects of difficult evacuation may precede or coexist with the proposed sequence. Microbial or immune feedback belongs to a later stage of the model [71,72] and cannot presently identify the initiating event.

## 8. Clinical and Research Implications

### 8.1. Clinical Interpretation

The model is not a diagnosis. Persistent constipation, incomplete evacuation, repeated straining, or disproportionate meal-related distension may justify task-specific functional assessment after childbirth. Relevant measures include pressure generation, abdominal accommodation, and outlet relaxation, rather than IRD or injury alone. Breath holding, excessive bracing, doming, pelvic pressure, urinary symptoms, or difficulty relaxing the pelvic floor can identify tasks requiring examination. These signs do not independently define a diagnostic phenotype.

Medication, diet and hydration, pain, activity, perineal or surgical injury, lactation-related routines, and pre-existing gastrointestinal disease remain competing explanations. Self-reported exercise does not verify task performance. Repeated cross-sectional postpartum samples show changes in reported physiotherapy use and exercise practices, although correct performance remained incomplete; they are not a six-year within-woman cohort or an instrumental assessment of coordination [94].

Intervention claims should remain outcome specific: non-postpartum biofeedback normalized transit in verified dyssynergia [58] and corrected abdominophrenic dyssynergia with distension [44]. A 2024 postpartum trial improved symptoms and stool form in both rehabilitation arms, with additional symptoms and quality-of-life benefit from TTNS [53]. A 2026 TTNS-plus-rehabilitation trial reported symptoms, sEMG, and manometric changes, with statistical and measurement inconsistencies [54]. The shared rehabilitation prevents attribution to stabilization alone; the 2025 postural programme improved type I pelvic-floor strength and urinary leakage, but not PFDI-20 or CRADI-8 [55]. This separates continence from colorectal–anal benefit, while the small sample and internally inconsistent enrollment reporting further limit interpretation [55]. Women-focused physiotherapy literature and postpartum PFMT meta-analysis support modifiable pelvic-floor outcomes [95,96], but no postpartum stabilization-targeted intervention identified here jointly demonstrated the proposed sequence from corrected mechanics through bowel function to intestinal and host-signal changes. Nutritional trials have assessed microbial and inflammatory outcomes independently of stabilization [83,84].

### 8.2. Research Design to Test the Model

A direct test should be longitudinal, with repeated within-woman measurements beginning before persistent dysfunction is established and extending beyond 6–8 weeks postpartum. Synchronizing mechanical function, bowel phenotype, and intestinal sampling within the same time windows is essential for temporal ordering.

Testing the pressure–outlet pathway requires task-specific measurement of diaphragm and abdominal-wall behavior, pressure generation/transmission, and pelvic-floor or anal relaxation, with IRD recorded separately. Bowel phenotyping should include symptoms, stool characteristics, and, where feasible, objective evacuation and transit. Evaluation of the respiratory-mechanics pathway requires direct measurement of visceral displacement and/or abdominal accommodation; a prespecified fluid-transport parameter is needed only when that arm is tested. Diaphragm excursion alone provides insufficient information about bowel mechanics.

Microbial profiling and fecal metabolites are informative only alongside stool form/transit and contemporaneous diet. Fiber and other fermentable substrates, lactation, medications, delivery-related care, pain, activity, adiposity, parity, and pre-existing bowel symptoms should be tracked longitudinally. A mechanism-focused intervention should change the verified mechanical deficit first; subsequent effects on evacuation, transit, and intestinal readouts would then test mediation rather than simple coexistence.

## 9. Conclusions

The proposed abdominopelvic-to-gut model integrates postpartum stabilization, bowel function, and nutritional interpretation through two candidate mechanical pathways with unequal support. Route A has the closer human physiological foundation and selected postpartum anorectal and symptom evidence, but its complete stabilization–evacuation–transit sequence has not been established after childbirth. For Route B, respiratory displacement and accommodation are measurable outside the postpartum setting; postpartum effects on intrinsic motility, transit, perfusion, or fluid transport remain hypotheses. Transit and stool residence provide a plausible bridge to luminal and microbial changes, with diet, lactation, medication, and other exposures acting as time-varying modifiers or independent causes. No identified study demonstrates the full temporal causal chain or subsequent recovery feedback. Structural findings such as IRD and birth-related injury remain complementary to functional measurement. The present contribution is a testable integrated framework for defined postpartum subgroups.

## Figures and Tables

**Table 1:** Evidence-directness summary for individual links in the proposed abdominopelvic-to-gut model.

Link	Evidence Available and Directness	Postpartum Specificity	Interpretive Boundary
Task-specific stabilization mechanics and pressure transmission	Indirect human physiology and case evidence: Diaphragmatic displacement and IAP depend on abdominal-wall compliance [20,21]; Abdominal-wall tension also correlates with IAP during clinical tests [26]. Postpartum respiratory and abdominal tasks show task-dependent pelvic-floor responses [27,28].	Postpartum respiratory/abdominal-task ultrasound shows altered multicomponent behavior [27,28]. Vaginal birth increases perineal-body/anorectal-junction mobility [29]; complete puborectalis avulsion reduced passive stiffness, strength, contraction speed, and endurance without between-group bowel-symptom differences [49].	Functional behavior cannot be inferred from IRD or a single pressure value. Ultrasound movement/thickening is not pressure or force; the case and nulliparous MRI are not postpartum bowel evidence.
Respiratory bowel displacement and abdominal accommodation	Indirect human evidence: respiratory small bowel/colon motion can be separated from intrinsic motility on MRI [40]. Gas-provocation and paired-CT studies measure accommodation [42,43]; biofeedback reduced distension symptoms [44], without establishing transit change.	No postpartum study measured respiratory bowel displacement/accommodation against bowel outcomes.	These measurements address displacement and accommodation; intrinsic motility and transit are separate outcomes.
Pressure-dependent fluid movement	Indirect human proxies: breathing-related vena-cava collapsibility and thoracic-duct diameter [45,46]; animal evidence: rabbit experiments show pressure- and frequency-dependent peritoneal drainage [47].	No postpartum study relates these phenomena to bowel outcomes.	Human diameter/collapsibility does not measure volumetric flow. Postpartum intestinal perfusion, local tissue-fluid exchange, motility, and transit require their own measurements.
Defecatory pressure–outlet coordination	Indirect human physiological and intervention evidence: Defecation requires coordinated abdominal pressurization and outlet relaxation [22,23]. MRI–manometry and ultrasound–manometry identify inadequate rectal pressure, insufficient anal relaxation, and abdomino-anal dyscoordination in defecatory disorders [24,25]. Biofeedback can normalize transit in selected non-postpartum dyssynergia [58].	Direct postpartum component evidence: selected manometric values [52,54] and symptoms after bundled rehabilitation ± TTNS [53,54]. Wu response counts do not confirm claimed significance [54]. Postural versus Kegel rehabilitation had no between-group CRADI-8 benefit [55].	Bundled interventions support modifiability of selected bowel, pelvic-floor, or continence outcomes. Stabilization was not isolated as the mediator, and transit was not tested. Small samples, attrition, and reporting inconsistencies limit inference; a postpartum-mediated sequence is not established.
Transit and intestinal environment	Indirect human evidence: stool form/transit associate with microbial composition and metabolites [60,61,62], with null adjusted associations also reported [63]. Massage shortened transit in three non-postpartum studies within a 23-study meta-analysis (*I*^2^ = 65%) [59].	No equivalent postpartum multilevel cohort.	Association does not establish initiation by stabilization. Higher richness is not necessarily beneficial. No postpartum temporal mechanics–transit–microbiota sequence is demonstrated.
Barrier, metabolites, and inflammation	Indirect biological and methodological evidence: SCFAs have epithelial, immune, and metabolic actions [68,69]; barrier markers have assay-specific limits [70]. Postpartum blood inflammation is non-specific; muscle-feedback causality is mainly animal evidence [8,71,72].	No postpartum study links the full functional sequence to metabolite exposure, barrier-related signaling, inflammation, and recovery.	These are candidate downstream mediators or feedback signals; an isolated biomarker cannot identify gut origin or causal direction.

Note: SCFAs, short-chain fatty acids; IAP, intra-abdominal pressure; IRD, inter-recti distance; MRI, magnetic resonance imaging; CT, computer tomography; TTNS, transcutaneous tibial nerve stimulation; sEMG, surface electromyography; CRADI-8, Colorectal-Anal Distress Inventory-8; LBP, lipopolysaccharide-bring protein; BMI, body mass index.

**Table 2:** Selected representative human studies of the postpartum maternal gut microbiome and its nutritional, lactation-related, metabolic, and clinical modifiers.

Study and Design	Population, Timing, and Method	Direct Contribution	Critical Limitation
Aparicio et al. [14] Longitudinal nutritional cohort	793 women; pregnancy and postpartum dietary assessment; nutrient intake compared with recommendations	Fiber and vitamins E and C intake decreased from pregnancy to postpartum, demonstrating that microbial substrate exposure changes across the sampling window.	No stool, bowel-function, transit, or microbiome outcome; dietary intake was self-reported.
Fenton et al. [15] Longitudinal cohort	337 women with complete paired dietary data from 2397 baseline respondents; third trimester and 1 year postpartum; food-frequency questionnaire and Australian Recommended Food Score	Diet quality remained low (34.0 vs. 34.8 of 73 points), while several food-group and nutrient intakes changed by one year postpartum.	No stool or microbiome measurement; a single late postpartum assessment cannot represent shorter-term dietary variation. Complete-case selection and self-reported food intake limit generalizability; supplements were not included, and a low score does not prove biochemical deficiency.
Carrothers et al. [16] Longitudinal observational study	20 healthy lactating women; 2 days–6 months postpartum; repeated diet records and 16S rRNA sequencing	Community structure was relatively stable; reported macro- and micronutrient intakes correlated with several major taxa.	Small, lactating-only cohort; correlated exposures and multiple comparisons; no causal inference.
Cortés-Martín et al. [77] One-year longitudinal cohort	40 women; 3 weeks and 4, 6, and 12 months postpartum; urolithin metabotyping and 16S rRNA sequencing	Microbial and anthropometric trajectories differed by functional ellagitannin-metabolism phenotype.	No dietary intervention or pre-pregnancy baseline; metabotype–recovery directionality unresolved.
Haddad et al. [17] Cross-sectional cohort	24 mother–infant dyads at 6 weeks postpartum; exclusive versus partial human-milk feeding, with all mothers lactating; maternal fecal 16S rRNA sequencing	Maternal Shannon group contrast was not significant (*p* = 0.06); separately reported Shannon and Sørensen model associations were BMI-sensitive. Bray–Curtis did not differ. Infant findings are distinct.	Small cross-sectional sample, BMI imbalance, self-reported feeding and ambient sample mailing. Main methods were verified; supplementary model tables were not checked in this revision, so precise adjusted estimates require author verification.
Selma-Royo et al. [76] Prospective cohort	167 mothers; first 4 months postpartum; delivery mode, cortisol, weight recovery, and 16S rRNA sequencing	Delivery-mode-related microbial profiles co-occurred with stress and weight-recovery differences.	Delivery, perioperative antibiotics, stress, and metabolic recovery cannot be cleanly separated.
Chalifour et al. [73] Longitudinal observational study	105 Latina mothers; 1 and 6 months postpartum; weight change, diet/activity measures, and 16S rRNA sequencing	Postpartum weight gain, but not weight loss, accompanied changes in diversity, taxa, and network structure.	No pre-pregnancy baseline; microbial change may be a cause, consequence, or correlate of weight trajectory.
Happel et al. [84] Randomized controlled trial	45 Lactating South African mothers randomized; fermented grain intervention from 4 to 10 weeks postpartum; dietary data, stool microbiota, nutritional and inflammatory measures	Live-culture fermented grain increased maternal gut alpha-diversity over the intervention and was associated with distinct microbial, nutritional, and inflammatory signatures.	Food-specific intervention in a particular nutritional and regional context; no abdominopelvic, evacuation, or transit outcomes. Attrition and the small sample reduce precision; microbial change is not itself clinical benefit.
Deng et al. [83] Randomized controlled trial	152 selected mother–infant dyads within a randomized trial; pregnancy, 1–2 and 5–6 months postpartum (105 available at the last maternal sampling); balanced energy–protein supplementation and shotgun metagenomics	Direct evidence that a multinutrient intervention can alter maternal microbial diversity, taxa, and functions.	Multicomponent intervention in a nutritionally vulnerable rural population; limited transportability to other settings. Microbiome-substudy selection and later sample loss limit transportability.

Note: BMI, body mass index; 16S rRNA, 16S ribosomal RNA gene.

**Table 3:** Interpretive boundaries for downstream signals and possible feedback within the postpartum abdominopelvic-to-gut model.

Candidate Signal	Evidence Relevant to the Model	Permissible Inference
SCFAs	Established microbial metabolites with epithelial, immune, and metabolic actions [68,69]; fecal patterns differed by transit and dyssynergic-defecation phenotype in one non-postpartum cohort [62]. Fecal concentration integrates production, cross-feeding, absorption, stool water, and transit.	Useful downstream signal, but fecal concentration does not directly measure production, absorption, systemic exposure, or benefit.
Bile-acid and tryptophan pathways	Established microbial–host signaling routes [86,87], but maternal postpartum evidence is insufficient.	Exploratory pathways; direction and relevance to postpartum functional recovery remain undefined.
Barrier function and microbial exposure	Intestinal permeability is biologically plausible [88], but no validated postpartum trajectory or abdominopelvic link exists. Sugar-recovery, protein, zonulin, and circulating LPS markers have distinct limitations [70,89,90,91].	Potential mediator; no isolated marker can establish gut-derived barrier failure.
Systemic inflammation	Direct postpartum component evidence: time/parity-related blood markers [8] and injury-related inflammation [92]. Indirect non-postpartum evidence: cross-sectional richness–stage associations lost significance after age adjustment, while selected taxon–stage associations persisted. Higher LBP and tissue inflammatory-cell infiltration came from unadjusted group comparisons; barrier failure was not demonstrated [93].	Non-specific contextual or mediating signal; gut origin and temporal direction require independent evidence.
Feedback to abdominopelvic recovery	Immune regulation of muscle repair is established, while microbiota–muscle causality is supported mainly by animal models [71,72].	Biologically plausible feedback pathway, but direct postpartum pelvic-floor, abdominal-wall, fascial, or neural evidence is lacking.

Note: SCFAs, short-chain fatty acids; LBP, lipopolysaccharide-bind protein; LPS, lipopolysaccharide.

## Data Availability

No new primary data were created or analyzed in this critical narrative review. The documented search strategies, evidence chart, and preliminary methodological appraisal are provided in the Supplementary Materials. Data sharing is not applicable to this article.

## Supplementary Materials

The following supporting information can be downloaded at: https://www.mdpi.com/article/10.3390/nu18213704/s1, Figure S1: Adapted PRISMA 2020 flow; Table S1: Search strategy; Table S2: Characteristics, Key Findings, and Methodological Limitations of the 40 Included Reports; Table S3: Item-Level Methodological Appraisal Matrices for Included Primary Studies.
