# Supplementary material for: Postpartum Abdominopelvic Stabilization Dysfunction and Bowel Function: A Proposed Mechanistic Model Linking Pressure Control, Visceral Mechanics, Transit, and the Intestinal Environment—A Critical Narrative Review
Source: Nutrients. 2026 Nov 8;18(21):3704. doi: 10.3390/nu18213704 (PMC13765409; doi:10.3390/nu18213704)
Supplement: Supplementary file 1 [file nutrients-18-03704-s001.zip › nutrients-4586445-supplementary.pdf]

Supplementary Figure S1

Adapted PRISMA 2020 flow

Records Identified:382

PubMed/MEDLINE: 91 Scopus: 187 Web of Science: 104

Duplicates removed:173

Reports sought for retrieval: 57

Report not retrieved: 10

Full-text exclusions: 7

Report retained:40

Core : 12 Supporting: 28

Supplementary Table S1

Search strategy:

PubMed/MEDLINE Recorded result count: 91.

(("Postpartum Period"[mh:noexp] OR postpartum[Title/Abstract] OR postnatal[Title/Abstract] OR puerperium[Title/Abstract] OR "after childbirth"[Title/Abstract]) AND ("pelvic floor"[Title/Abstract] OR "pelvic floor muscle*"[Title/Abstract] OR diaphragm*[Title/Abstract] OR "abdominal wall"[Title/Abstract] OR "abdominal muscle*"[Title/Abstract] OR abdominopelvic[Title/Abstract] OR lumbopelvic[Title/Abstract] OR "core stabil*"[Title/Abstract] OR "intra-abdominal pressure"[Title/Abstract]) AND (("gastrointestinal motility"[MeSH Terms] OR "intestinal motility"[Title/Abstract] OR "colonic motility"[Title/Abstract] OR "intestinal transit"[Title/Abstract] OR "colonic transit"[Title/Abstract] OR peristalsis[Title/Abstract] OR constipation[Title/Abstract] OR defecat*[Title/Abstract] OR "obstructed defecation"[Title/Abstract] OR "dyssynergic defecation"[Title/Abstract] OR "bowel dysfunction"[Title/Abstract]) OR (bloating[Title/Abstract] OR "abdominal distension"[Title/Abstract] OR "abdominal discomfort"[Title/Abstract] OR "functional gastrointestinal disorder*"[Title/Abstract] OR "disorders of gut-brain interaction"[Title/Abstract] OR "irritable bowel syndrome"[Title/Abstract])))

Scopus Recorded result count: 187.

TITLE-ABS-KEY((postpartum OR postnatal OR puerperium OR "after childbirth") AND ("pelvic floor" OR "pelvic floor muscle*" OR diaphragm* OR "abdominal wall" OR "abdominal muscle*" OR abdominopelvic OR lumbopelvic OR "core stabil*" OR "intra-abdominal pressure") AND (("gastrointestinal motility" OR "intestinal motility" OR "colonic motility" OR "intestinal transit" OR "colonic transit" OR peristalsis OR constipation OR defecat* OR "obstructed defecation" OR "dyssynergic defecation" OR "bowel dysfunction") OR (bloating OR "abdominal distension" OR "abdominal discomfort" OR "functional gastrointestinal disorder*" OR "disorders of gut-brain interaction" OR "irritable bowel syndrome")))

Web of Science Core Collection Recorded result count: 104.

TS=((postpartum OR "post-partum" OR postnatal OR puerperium OR "after childbirth") AND ("pelvic floor" OR "pelvic floor muscle*" OR diaphragm* OR "abdominal wall" OR "abdominal muscle*" OR abdominopelvic OR lumbopelvic OR "core stabil*" OR "intra-abdominal pressure") AND (("gastrointestinal motility" OR "intestinal motility" OR "colonic motility" OR "intestinal transit" OR "colonic transit" OR peristalsis OR constipation OR defecat* OR "obstructed defecation" OR "dyssynergic defecation" OR "bowel dysfunction") OR (bloating OR "abdominal distension" OR "abdominal discomfort" OR "functional gastrointestinal disorder*" OR "disorders of gut-brain interaction" OR "irritable bowel syndrome")))

Eligibility record

Eligibility criteria covered primary clinical studies, randomized and nonrandomized interventions, prospective/retrospective cohorts, cross-sectional reports, and relevant reviews or consensus addressing postpartum or childbirth-related abdominopelvic/pelvic-floor/anorectal function and bowel/GI outcomes. Exclusions covered exclusively urinary/sexual/prolapse questions without a relevant GI component; acute complications unrelated to functional physiology; neonatal/pediatric reports; animal studies in the structured postpartum search; isolated pharmacological/anesthetic interventions; and unrelated GI disorders. S2–S3 present the included, full-text-excluded and not-retrieved reports.

The included set also contains morphology, continence and modifier reports without direct bowel physiology; S2 labels their indirect or contextual contributions. The epidural-exposure cohort S2-24 is a negative modifier comparison despite the exclusion of isolated anesthetic interventions; its contextual role is explicit. These modifier reports are not counted as direct tests of the bowel pathway. Animal and technical sources cited for mechanistic context lie outside the structured-search set.

Supplementary Table S2 Characteristics, Key Findings, and Methodological Limitations of the 40 Included Reports

Appraisal codes: A = primary study included in the methodological appraisal; W = secondary review or consensus not assessed using primary-study checklists. Codes separated by a vertical bar (|) indicate appraisal status followed by study design (e.g., A | R = appraised randomized controlled trial; A | C = appraised cohort study). R = randomized controlled trial; C = cohort study; X = analytical cross-sectional study; Q = quasi-experimental study; P = prevalence study; K = case–control study. These codes indicate appraisal status and study design, not methodological quality or evidence certainty.

Report and sample or timing	Exposure and measurements	Bowel finding and permissible contribution	Evidence relation and family	Appraisal and methodological limits
S2-3 | Main reference 22<br>Impact of pregnancy and parturition on the anal sphincters and pelvic floor (2007)<br>Clinical narrative review<br>Pregnancy and postpartum literature<br>https://doi.org/10.1016/j.bpg.2007.06.001	Anal sphincter, pelvic-floor, defecation, and obstetric physiology	Defecation physiology and pregnancy/postpartum context; integrated diaphragm–abdominal wall–pelvic floor–IAP function.	Indirect mechanistic or intervention support<br>Core<br>No report family recorded	W | Not a primary-report checklist<br>Secondary synthesis; no connector-specific causal estimate. Secondary source, not a new primary experiment. Not entered into the primary-report methodological appraisal.
S2-5 | S2-only report<br>Fecal incontinence in pregnancy and post partum (2009)<br>Cohort report<br>240 participants at entry; 151 at 1 year. Followed in pregnancy and postpartum; bowel-accident item in a secondary analysis of trial data.<br>https://doi.org/10.1016/j.ijgo.2009.04.018	Fecal-incontinence symptoms over time	Longitudinal continence-symptom context. Few bowel-accident events; nonsignificant treatment comparisons do not establish equivalence.	Context, burden, trajectory, or modifier<br>Supporting<br>No report family recorded	A | C<br>Secondary analysis of trial participant data: 240 at entry, 151 at one year. Single bowel-accident question; few events, unadjusted comparisons and unexplained follow-up losses. Not a review.
S2-6 | Main reference 29<br>Mobility of the perineal body and anorectal junction before and after childbirth (2012)<br>Cohort report<br>200 enrolled; 156 paired datasets. Ultrasound at 36–38 gestational weeks and 3–6 months postpartum.<br>https://doi.org/10.1007/s00192-012-1672-8	Anorectal-junction and perineal-body mobility	Perineal-body and anorectal-junction mobility increased after birth (both $p<0.001$). This is displacement, not evacuation efficiency or intestinal transit.	Indirect mechanistic or intervention support<br>Supporting<br>No report family recorded	A | C<br>200 enrolled; 157 followed, 156 paired datasets. Standardized blinded ultrasound and repeatability assessment; no multivariable control of obstetric confounding; reasons for incomplete follow-up insufficiently resolved.
S2-12 | S2-only report<br>Obstructed Defecation Syndrome After Delivery Trauma (2015)<br>Quasi-experimental report<br>30 consecutive surgical patients; mean age 54.4 years. Historical childbirth exposure, not a defined early postpartum cohort.<br>https://doi.org/10.5812/traumamon.25611	Obstructed-defecation symptoms and delivery-trauma context	Clinical obstructed-defecation context after historical childbirth exposure. STARR-treated, selected older patients do not establish a causal early postpartum mechanism.	Non-early-postpartum clinical context; historical childbirth exposure<br>Supporting<br>No report family recorded	A | Q<br>30 consecutive surgical patients; mean age 54.4 years, not a defined early postpartum cohort. All underwent STARR; no untreated comparator, no confounder adjustment for historical birth comparisons.

S2-13 | Main reference 10<br>Pregnancy and postpartum bowel changes: constipation and fecal incontinence (2015)<br>Clinical review<br>Pregnancy and postpartum literature<br>https://doi.org/10.1038/ajg.2015.76	Constipation, fecal incontinence, and postpartum physiology	Pregnancy/postpartum bowel review with integrated defecatory physiology.	Direct postpartum bowel/functional evidence<br>Core<br>No report family recorded	W | Not a primary-report checklist<br>Secondary synthesis; heterogeneous underlying evidence. Secondary source, not a new primary experiment. Not entered into the primary-report methodological appraisal.
S2-14 | S2-only report<br>Early pelvic floor muscle training after obstetrical anal sphincter injuries for the reduction of anal incontinence (2016)<br>Quasi-experimental report with historical controls<br>211 respondents of 230 identified after OASI: 102 early-plus-standard and 109 standard rehabilitation; 167 per protocol. Median follow-up 16 versus 24 months.<br>https://doi.org/10.1016/j.ejogrb.2016.01.025	Early PFMT; anal-incontinence outcome	Early-plus-standard physiotherapy showed a symptom signal after OASI. Historical allocation and unequal follow-up prevent attribution to a single stabilization component.	Indirect mechanistic or intervention support<br>Supporting<br>No report family recorded	A | Q<br>Historical-control comparison after OASI: 230 identified, 211 respondents (102 early plus standard, 109 standard); 167 per-protocol. Injury severity differs; median follow-up 16 versus 24 months is unadjusted. Modified questionnaire reliability and loss patterns are incompletely documented; several text/table values conflict.
S2-15 | S2-only report<br>Continuous recording of intrarectal pressures during the second phase of labour: correlations with postpartum pelvic floor complaints. A biomechanical-clinical study (2017)<br>Cohort report<br>99 enrolled; 60 followed (43 spontaneous and 17 forceps births). Labour pressure recording; postpartum complaints at approximately 14 ± 6 months.<br>https://doi.org/10.1007/s00192-016-3249-4	Continuous intrarectal pressure during labour; postpartum complaints	Intrapartum pressure exposure; indirect biomechanical evidence, low weight for bowel axis.	Indirect mechanistic or intervention support<br>Supporting<br>No report family recorded	A | C<br>99 enrolled, 60 followed (approximately 39% loss).<br>Pressure recordings susceptible to movement/baseline artefact; delivery-stratified comparisons do not resolve confounding; wide follow-up timing.
S2-17 | Main reference 49<br>Pelvic floor morphometry and function in women with and without puborectalis avulsion in the early postpartum period (2017)<br>Analytical cross-sectional report<br>52 women with or without complete puborectalis avulsion, assessed at approximately 3 months postpartum. Chronic constipation and partial avulsion excluded.<br>https://doi.org/10.1016/j.ajog.2016.11.1049	Pelvic-floor morphometry/function, avulsion, and symptoms	Complete avulsion accompanied lower passive stiffness, strength, contraction speed and endurance, without a between-group bowel-symptom difference. Constipation was excluded.	Indirect mechanistic or intervention support<br>Core<br>No report family recorded	A | X<br>52 women at one postpartum visit; selected for avulsion risk. Blinded structural/functional assessment, but no multivariable confounder control. Chronic constipation excluded; partial avulsion excluded.
S2-18 | S2-only report<br>Pregnancy-Associated Pelvic Floor Health	Antenatal education plus PFME; CRADI-8 symptoms and CRAIQ-7	CRAIQ-7 bowel-related quality-of-life signal; CRADI-8 symptom	Indirect mechanistic or intervention support	A | R<br>100 randomized; 74 provided

Knowledge and Reduction of Symptoms: The PREPARED Randomized Controlled Trial (2018)<br>Randomized trial<br>100 randomized in pregnancy; 74 supplied postpartum data at 6 weeks. Education/PFME workshop versus usual care.<br>https://doi.org/10.1016/j.jogc.2017.10.022	impact on quality of life are separate outcomes	difference was not significant (p=0.247). Do not describe this as fewer bowel symptoms.	Supporting<br>No report family recorded	postpartum data. No participant masking; concealment incompletely described. Bowel outcomes secondary with multiple comparisons, no bowel-specific power. Assigned-group available-case analysis.
S2-19 | Main reference 32<br>Anal incontinence and unrecognized anal sphincter injuries after vaginal delivery- a cross-sectional study in Norway (2020)<br>Analytical cross-sectional report<br>207/396 questionnaire respondents; 97 ultrasound examinations. Mean 14 months after vaginal birth with perineal suturing; recognized OASI excluded.<br>https://doi.org/10.1186/s12905-020-00989-5	Anal-incontinence questionnaire and sphincter-injury assessment	At a mean 14 months after vaginal birth, fecal urgency and flatus leakage were reported and previously unrecognized third-degree injury was identified in a subset. Supports closure/injury manifestations of PFM dysfunction, not an evacuation or transit mechanism.	Context, burden, trajectory, or modifier<br>Supporting<br>No report family recorded	A | X<br>207/396 questionnaire responses; only 97 ultrasound assessments. Perineal-suture sample; recognized OASI excluded. Standardized symptom/ultrasound measures; no adjusted injury-symptom model.
S2-20 | Main reference 6<br>Pelvic floor dysfunction symptoms and risk factors at the first year of postpartum women: a cross-sectional study (2020)<br>Analytical cross-sectional report<br>408/510 volunteers at purposively selected health centres; 3–12 months postpartum.<br>https://doi.org/10.1080/10376178.2020.1749099	Pelvic-floor symptom questionnaire including bowel symptoms	Postpartum PFD symptom epidemiology including constipation/flatulence/fecal incontinence.	Context, burden, trajectory, or modifier<br>Supporting<br>No report family recorded	A | X<br>408/510 volunteers from purposively selected health centers. Investigator questionnaire has expert review and small pilot, but limited reliability evidence. Regression for several PFD outcomes, not constipation; only 12 FI events.
S2-21 | Main reference 36<br>Association Between Measures of Trunk Recovery 5 to 10 Weeks Postpartum and Pelvic Floor Support and Symptoms 1 Year Postpartum in Primiparas Delivered Vaginally (2021)<br>Cohort report<br>624/825 MAP participants at follow-up. Trunk recovery at 5–10 weeks; support/symptoms at 1 year.<br>https://doi.org/10.1097/SPV.0000000000000953	Trunk recovery tests; pelvic support and symptom burden	Early trunk/IAP measures were assessed against one-year support and symptoms. Lifting IAP and trunk endurance did not predict the later outcomes; no pressure–outlet or transit measurement.	Indirect mechanistic or intervention support<br>Supporting<br>MAP cohort	A | C<br>624/825 completed follow-up; differential nonresponse. Validated pressure sensor, trunk tests and adjusted models including baseline status; no demonstrated strategy correcting whole-participant attrition.
S2-22 | Main reference 37<br>Do Measures of Muscular Fitness Modify the Effect of Intra-abdominal Pressure on Pelvic Floor Support in Postpartum Women? (2021)<br>Analytical cross-sectional report within MAP<br>593/825 MAP participants at 1 year; 138/593 missing pelvic-floor force owing to device	Muscular fitness, IAP exposure, and pelvic-floor support	Assessed muscular fitness as a modifier of the IAP–support relation, without consistent modification. Cross-sectional at 1 year; constipation was a covariate/loading factor, not measured transit.	Indirect mechanistic or intervention support<br>Supporting<br>MAP cohort	A | X<br>593/825 at one year; pelvic-floor force missing in 138/593 owing to device interruption. Validated measures and conceptual-model adjustment; selection and

interruption. Cross-sectional one-year analysis.<br>https://doi.org/10.1097/SPV.0000000000000901				reduced interaction precision remain.
S2-23 | Main reference 79<br>Early postpartum physical activity and pelvic floor support and symptoms 1 year postpartum (2021)<br>Cohort report<br>611/825 MAP participants followed to 1 year; accelerometry at 2–3 and 5–6 weeks postpartum.<br>https://doi.org/10.1016/j.ajog.2020.08.033	Accelerometer-derived MVPA; one-year support and symptom outcomes	MAP cohort report: early postpartum MVPA showed mainly null or nonlinear associations with one-year pelvic-floor support and symptoms. Activity is a modifier; accelerometry does not measure task-specific stabilization.	Context, burden, trajectory, or modifier<br>Supporting<br>MAP cohort	A | C<br>611/825 one-year follow-up; different nonresponder characteristics. Accelerometry quality checks, DAG-based adjustment and covariate imputation; these do not correct missing whole-participant outcomes.
S2-24 | Main reference 81<br>Effect of Epidural Analgesia on Pelvic Floor Dysfunction at 6 Months Postpartum in Primiparous Women: A Prospective Cohort Study (2021)<br>Cohort report<br>150 analyzed after 208 consented; 74 epidural recipients. Outcomes at 6 months postpartum; exposure self-selected.<br>https://doi.org/10.1016/j.esxm.2021.100417	Epidural exposure; PFDI-20, palpation, and pelvic-floor sEMG	Other PFD symptoms and muscle function, including bowel symptoms, did not differ significantly at 6 months; not an equivalence trial.	Context, burden, trajectory, or modifier<br>Supporting<br>No report family recorded	A | C<br>150 analyzed after 208 consented; 35 missed follow-up after delivery exclusions. Analgesia self-selected. Adjustment reported for dyspareunia, not bowel endpoint; validated questionnaire items but no motility measure.
S2-25 | Main reference 11<br>Obstetric risk factors for anorectal dysfunction after delivery: a systematic review and meta-analysis (2021)<br>Systematic review and meta-analysis<br>Postpartum anorectal studies eligible for systematic synthesis<br>https://doi.org/10.1007/s00192-021-04723-z	Obstetric exposures and postpartum anorectal dysfunction	Systematic review showing evidence gap for postpartum constipation and limitations of obstetric-risk explanation.	Direct postpartum bowel/functional evidence<br>Core<br>No report family recorded	W | Not a primary-report checklist<br>Sparse and heterogeneous evidence; highlights rather than resolves the gap. Secondary source, not a new primary experiment. Not entered into the primary-report methodological appraisal.
S2-28 | S2-only report<br>Self-reported symptoms in women with diastasis rectus abdominis: A systematic review (2021)<br>Systematic review<br>Studies of women with diastasis recti abdominis<br>https://doi.org/10.1016/j.jogoh.2020.101995	DRA morphology and self-reported symptoms	DRA symptom systematic review; contextual boundary for morphology versus function.	Indirect mechanistic or intervention support<br>Supporting<br>No report family recorded	W | Not a primary-report checklist<br>Self-reported symptoms and DRA morphology; function not established. Secondary source, not a new primary experiment. Not entered into the primary-report methodological appraisal.
S2-29 | S2-only report<br>Trajectories of Pelvic Floor Symptoms and Support After Vaginal Delivery in Primiparous Women Between Third Trimester and 1 Year Postpartum (2021)	Validated pelvic-floor symptom and support trajectories	Longitudinal trajectories of postpartum pelvic-floor symptoms/support; recovery heterogeneity.	Context, burden, trajectory, or modifier<br>Supporting<br>MAP cohort	A | C<br>597 complete questionnaire trajectories; 44 lack one-year POP-Q. Differential retention; missing bother scored absent.

Cohort report<br>597 complete MAP questionnaire trajectories; 44 missing one-year POP-Q. Third trimester to 1 year postpartum.<br>https://doi.org/10.1097/SPV.0000000000001068				Validated instruments, DAG adjustment, multiplicity correction and sensitivity E-values; no full correction of attrition.
S2-30 | S2-only report<br>Which symptoms of pelvic floor dysfunction does physiotherapy improve after an OASI? (2021)<br>Quasi-experimental report<br>131 women after OASI at baseline within 6 weeks; 96 followed at 3–6 months. Uncontrolled physiotherapy follow-up.<br>https://doi.org/10.1016/j.ejogrb.2021.07.048	Post-OASI physiotherapy and anorectal symptoms	Flatus leakage and ability to defer a bowel motion improved within the observed cohort; fecal incontinence did not show a corresponding significant change. No untreated comparison.	Indirect mechanistic or intervention support<br>Supporting<br>No report family recorded	A | Q<br>Uncontrolled pre/post physiotherapy; 131 baseline, 96 follow-up. Natural recovery and co-interventions unresolved; treatment adherence/reliability unclear; reported unpaired tests for repeated observations raise concern.
S2-31 | Main reference 80<br>Pelvic Floor Support and Symptoms 1 Year Postpartum in Relation to Excess Body Habitus Before, During and After First Vaginal Birth (2022)<br>Cohort report<br>645/825 MAP participants at 1 year. Prepregnancy BMI, pregnancy gain and postpartum retention.<br>https://doi.org/10.1097/SPV.0000000000001150	BMI, gestational gain, retention; support and symptom outcomes	MAP cohort report: pre-pregnancy BMI, gestational gain, and weight retention were not associated with most one-year support or symptom outcomes, including anal incontinence and constipation. Defines a negative modifier boundary.	Context, burden, trajectory, or modifier<br>Supporting<br>MAP cohort	A | C<br>645/825 follow-up; pre-pregnancy weight self-reported and exploratory thresholds. Adjusted models and nonresponder comparison; no correction of participant attrition. Constipation defined using two symptom items.
S2-32 | S2-only report<br>Prevalence and severity of pelvic floor disorders in pregnant and postpartum women (2022)<br>Prevalence report; complementary X risk-factor appraisal<br>2007 anonymous questionnaires: 1048 during pregnancy and 959 within 6 weeks postpartum. Eight centres; not a paired longitudinal sample.<br>https://doi.org/10.1002/ijgo.14019	Validated PFD survey; prevalence and risk-factor analyses; contraction ability self-reported. No objective evacuation or transit test	Pregnancy and postpartum symptom prevalence, not an individual recovery trajectory. Self-reported contraction ability did not associate significantly with constipation (p=0.61) or fecal incontinence (p=0.16).	Context, burden, trajectory, or modifier<br>Supporting<br>No report family recorded	A | P<br>Cross-sectional anonymous survey: 2007 questionnaires, 1048 in pregnancy and 959 postpartum, eight centers. Invitation denominator and sampling method are unreported; possible repeated respondents cannot be linked. Validated symptom instrument; unadjusted risk-factor analyses and no control for index birth or lactation.
S2-33 | Main reference 9<br>An International Continence Society (ICS)/ International Urogynecological Association (IUGA) joint report on the terminology for the assessment and management of obstetric pelvic floor disorders (2023)<br>Consensus terminology report<br>Obstetric pelvic-floor terminology and	Definitions and outcome classification	ICS/IUGA terminology for obstetric pelvic-floor/anorectal dysfunction; definitions and outcome classification.	Direct postpartum bowel/functional evidence<br>Core<br>No report family recorded	W | Not a primary-report checklist<br>Terminology source; not causal evidence. Secondary source, not a new primary experiment. Not entered into the primary-report methodological appraisal.

assessment literature<br>https://doi.org/10.1007/s00192-022-05397-x				
S2-35 | Main reference 50<br>Association between functional constipation and vaginal wind in women at 6 weeks postpartum (2023)<br>Analytical cross-sectional report<br>377 women at 6 weeks postpartum. Functional constipation and vaginal wind with pelvic-floor sEMG/clinical data.<br>https://doi.org/10.1007/s00192-023-05619-w	Functional constipation, vaginal wind, and pelvic-floor sEMG	Functional constipation in 123/377 at 6 weeks; association with vaginal wind (OR 2.41, 95% CI 1.43–4.07). Vaginal wind is not an evacuation or intestinal gas/transit endpoint.	Direct postpartum bowel/functional evidence<br>Core<br>No report family recorded	A | X<br>377 women at six weeks. Confounder adjustment, propensity methods and imputation are strengths. Vaginal wind is a limited self-report measure; Rome IV chronicity includes antecedent pregnancy history.
S2-36 | Main reference 52<br>Association of delivery mode and number of pregnancies with anorectal manometry data in patients with postpartum constipation (2023)<br>Analytical cross-sectional report using retrospective clinic records<br>127 selected patients with persistent postpartum constipation; retrospective clinic records and anorectal manometry. Duration varied; no healthy comparator.<br>https://doi.org/10.1186/s12884-023-05480-1	Anorectal manometry and delivery history	Anorectal pressure differences by delivery history in a selected symptomatic sample. These alone do not diagnose dyssynergia or demonstrate transit delay.	Direct postpartum bowel/functional evidence<br>Core<br>No report family recorded	A | X<br>127 symptomatic clinic patients, retrospective records. Standardized manometry and adjustment for age, parity and duration; selected sample, residual obstetric/treatment confounding, no healthy comparator.
S2-37 | Main reference 56<br>Effect Of Six Weeks Of Whole-Body Vibration In Treatment Of Postnatal Constipation (2023)<br>Randomized trial<br>40 women with postnatal constipation; 6-week trial. WBV added to shared pelvic-floor/static-abdominal exercise and diet.<br>https://doi.org/10.47391/JPMA.EGY-S4-3	Whole-body vibration add-on to pelvic-floor/abdominal exercise and diet	Incremental WBV symptom/quality-of-life signal over common exercise and diet. No objective motility, transit or isolated stabilization mediator measured.	Direct postpartum bowel/functional evidence<br>Supporting<br>No report family recorded	A | R<br>40 women; computer-generated allocation in sealed envelopes, opacity/sequence unclear. No sham; assessor masking not clearly separated from allocation. Participant flow and adherence sparse; narrative significance contradicts reported p for treatment main effect.
S2-38 | Main reference 48<br>Fecal incontinence and associated pelvic floor dysfunction during and one year after the first pregnancy (2023)<br>Cohort report<br>898 POPRACT participants with at least one observation; about 694–699 one-year bowel responses. Pregnancy to 1 year postpartum.<br>https://doi.org/10.1111/aogs.14614	Fecal incontinence and associated pelvic-floor dysfunction	At 1 year: fecal incontinence 6% and obstructed defecation 28% among available responses. "Slow bowel transit symptoms" are symptom proxies, not a transit measurement.	Context, burden, trajectory, or modifier<br>Supporting<br>POPRACT cohort	A | C<br>898 provide at least one observation; about 694–699 provide one-year bowel data. Adjusted repeated-measure models include partial observations, but missing-at-random assumption/whole-participant attrition unresolved.
S2-39 | S2-only report<br>The Impact of Pelvic Floor Physical Therapy on	PFPT after OASI; bladder and bowel outcomes	Validated fecal-incontinence scores document symptoms.	Indirect mechanistic or intervention support	A | C<br>169/353 eligible women provided

Bladder and Bowel Function After Obstetric Anal Sphincter Injury (2023)<br>Cohort report<br>169/353 eligible women provided outcome surveys (47.9%); PFPT and non-PFPT groups after OASI. Baseline and 6-month symptom questionnaires.<br>https://doi.org/10.1097/SPV.0000000000001286		Within-group improvement is not proof of a between-group physiotherapy effect.	Supporting<br>No report family recorded	outcome surveys (47.9%). PFPT group had worse baseline symptoms. Attendance does not standardize dose; adherence regression does not adjust the bowel treatment effect.
S2-40 | Main reference 67<br>The TOR concept (training, operation, and rehabilitation) applied to a cohort of postpartum women with training-resistant symptomatic rectus diastasis: evaluation 1 year after surgery (2023)<br>Quasi-experimental report<br>71 selected women more than 1 year postpartum with training-resistant DRA/core instability. Training, surgery and rehabilitation; outcome 1 year after surgery.<br>https://doi.org/10.1093/bjsopen/zrac162	Training–operation–rehabilitation program; constipation, UI, and core function	Constipation fell from 21.1% to 1.4% in an uncontrolled training–operation–rehabilitation programme. No isolated surgery effect or stabilization mediation established.	Indirect mechanistic or intervention support<br>Supporting<br>No report family recorded	A | Q<br>71 selected women; surgery, preoperative training and rehabilitation bundled. No untreated comparator or repeated pretreatment measurements; bowel outcome is a simple self-report. Domain-specific response counts vary.
S2-41 | Main reference 53<br>Bilateral transcutaneous tibial nerve stimulation: a promising treatment for women with postpartum constipation: A randomized controlled trial (2024)<br>Randomized trial<br>56 randomized women with postpartum constipation. Abstract and methods disagree about assessment timing; one exact follow-up duration is not treated as verified.<br>https://doi.org/10.5114/areh.2024.144633	TTNS add-on; diaphragmatic breathing, abdominal training, constipation outcomes	Incremental TTNS effect over shared breathing/abdominal rehabilitation; shared exercises were not independently tested. No established transit effect.	Direct postpartum bowel/functional evidence<br>Core<br>No report family recorded	A | R<br>56 randomized; no sham for participants/providers. Independent randomization reported, but index-card concealment insufficiently described. Abstract and methods disagree about assessment timing; reliability/reporting precision incompletely resolved.
S2-42 | Main reference 5<br>Body image and pelvic floor dysfunction in pregnancy and postpartum: A prospective one-year follow-up cohort study (2024)<br>Cohort report<br>305 recruited; 208 (68.2%) returned the one-year questionnaire. Pregnancy to 1 year; pre-pregnancy symptoms recalled retrospectively.<br>https://doi.org/10.1111/1471-0528.17820	Body image and PFD symptoms including constipation and UI	Persistent, clustered symptoms and body-image associations; 89/208 reported constipation at 1 year. No stabilization, motility, microbiota or barrier measurements.	Context, burden, trajectory, or modifier<br>Supporting<br>No report family recorded	A | C<br>305 recruited; 208 returned the one-year questionnaire (68.2%). Responders differ in education, BMI and parity. Validated Dutch symptom scales and adjusted repeated-measures mixed models are strengths. Retrospective pre-pregnancy symptoms; follow-up reasons and detailed estimates in unavailable supplements need verification.

S2-43 | Main reference 51<br>Pelvic floor dysfunction in postpartum women: A cross-sectional study (2024)<br>Analytical cross-sectional report<br>845/1022 convenience recruits analyzed at 1 month postpartum. Gestational constipation history and postpartum resting sEMG.<br>https://doi.org/10.1371/journal.pone.0308563	Gestational constipation and postpartum resting pelvic-floor sEMG	Gestational constipation associated with abnormal resting postpartum sEMG (OR 1.553, 95% CI 1.022–2.359). Temporal direction and evacuation physiology were not tested.	Indirect mechanistic or intervention support<br>Supporting<br>No report family recorded	A | X<br>845 analyzed from 1022 convenience recruits. Adjusted models and specified protocol; sEMG cutoffs lack study-specific reliability confirmation. Multiple outcomes require exploratory interpretation.
S2-46 | S2-only report<br>Levator Ani Deficiency and Pelvic Floor Dysfunction 1 Year Postpartum: A Prospective Nested Case-Control Study (2025)<br>Case–control report<br>103 cases and 87 unmatched controls from POPRACT at 1 year. Prospective parent cohort, but nested case–control structural/symptom analysis.<br>https://doi.org/10.1111/1471-0528.18036	Levator ani deficiency, support, and symptom outcomes	Levator deficiency and PFD at 1 year; structural/functional boundary, interpreted apart from bowel causality.	Indirect mechanistic or intervention support<br>Supporting<br>POPRACT cohort	A | K<br>103 cases and 87 unmatched controls from POPRACT. Participation selected; symptom-defined recruitment and exploratory cutoffs. Blinded repeated ultrasound assessment and adjusted models are strengths; temporal exposure ordering not demonstrated.
S2-47 | Main reference 18<br>Postpartum Anorectal and Pelvic Floor Disorders: Evaluation, Treatment, and Prevention (2025)<br>Clinical review<br>Postpartum anorectal and pelvic-floor clinical literature<br>https://doi.org/10.1007/s11894-025-01000-7	Evaluation, treatment, prevention, and evidence gaps	Current postpartum anorectal/PFD clinical review; context, diagnosis, treatment, evidence gaps.	Direct postpartum bowel/functional evidence<br>Core<br>No report family recorded	W | Not a primary-report checklist<br>Clinical review; conclusions depend on heterogeneous primary evidence. Secondary source, not a new primary experiment. Not entered into the primary-report methodological appraisal.
S2-48 | S2-only report<br>Symptoms of pelvic floor dysfunctions during pregnancy and postpartum (2025)<br>Cohort report in separate pregnant and postpartum samples<br>Separate pregnant and postpartum cohorts. Postpartum observations n=65, 53 and 39 at 0–6, 7–24 and >24 weeks, respectively. Interviews initially in person, then by telephone.<br>https://doi.org/10.1186/s12884-024-07071-0	Longitudinal PFD symptoms including bowel domain	Describes symptom trajectories in separate pregnancy and postpartum samples. Main time-related differences concern urinary and sexual function; does not establish a continuous pregnancy-to-postpartum or mechanical-to-bowel sequence.	Context, burden, trajectory, or modifier<br>Supporting<br>No report family recorded	A | C<br>Separate pregnant and postpartum cohorts, not one pregnancy-to-postpartum sequence. Postpartum observations 65, 53 and 39; mode changes to telephone. Confounder adjustment and attrition handling insufficient.
S2-49 | Main reference 12<br>Transperineal Ultrasound Immediately After Birth to Predict Postpartum Anorectal Dysfunction: A Prospective Cohort Study (2025)<br>Cohort report<br>182 complete assessments from 329 initially	Immediate transperineal ultrasound and postpartum constipation	Constipation affected 43%; immediate structural ultrasound did not predict it, whereas constipation at term did (aOR 3.73, 95% CI 1.81–7.69). A structural	Direct postpartum bowel/functional evidence<br>Core<br>No report family recorded	A | C<br>182 complete assessments from 329 initially recruited after eligibility losses; six-week follow-up. Blinded ultrasound reader, validated symptom scales and

recruited, after eligibility and follow-up losses. Ultrasound immediately after birth; symptoms at 6 weeks. https://doi.org/10.1007/s00192-025-06344-2		null result does not test functional pressure control.		adjusted models; residual selection/attrition concerns.
S2-50 | Main reference 66<br>Improvement of Gastrointestinal Symptoms After Diastasis Recti Repair: A Prospective Study (2026)<br>Quasi-experimental report<br>80 symptomatic postpartum women undergoing lipoabdominoplasty/diastasis repair with additional procedures and postoperative care. Follow-up at least 6 months after surgery, mean 14 months (range 6–30).<br>https://doi.org/10.1007/s00266-025-05588-z	DRA repair; self-reported GI symptoms	Self-reported GI improvement after an uncontrolled bundled operation. Baseline constipation is inconsistent (50% versus 60%); neither figure is selected as definitive.	Direct postpartum bowel/functional evidence<br>Core<br>No report family recorded	A | Q<br>80 women; uncontrolled lipoabdominoplasty with additional procedures, binder and exercise. Unvalidated five-point symptom ratings; conflicting baseline constipation percentages (50% versus 60%).
S2-52 | S2-only report<br>Pelvic Floor Dysfunction in Postpartum Nepalese Women: An Observational Study (2026)<br>Prevalence report<br>747 primiparas at a Nepalese tertiary centre; telephone survey at 9–12 months postpartum.<br>https://doi.org/10.31729/jnma.v64i293.9297	Postpartum PFD prevalence and symptom domains	Postpartum PFD prevalence; contextual clinical burden if bowel domain reported.	Context, burden, trajectory, or modifier<br>Supporting<br>No report family recorded	A | P<br>747 primiparas, single tertiary center, telephone survey at 9–12 months. No validated Nepali instrument; expert translation and 10-person pilot only. Recruitment denominator/nonresponse insufficiently resolved; outcome denominators vary.
S2-54 | Main reference 33<br>Self-reported pelvic floor dysfunction 12 months after an obstetric anal sphincter injury in relation to maternal body mass index (2026)<br>Cohort report<br>6595 first vaginal births with OASI; 3457/6595 respondents (52%) at 12 months.<br>https://doi.org/10.1111/aogs.70171	BMI; anal and urinary incontinence, splinting, and other PFD symptoms	National OASI cohort: anal incontinence remained common at 12 months and did not vary by BMI, whereas urinary incontinence increased with BMI. Supports heterogeneous continence manifestations and modifier effects, not a stabilization pathway.	Context, burden, trajectory, or modifier<br>Supporting<br>No report family recorded	A | C<br>3457/6595 OASI respondents (52%). Registry exposure and validated symptom instrument; baseline complaints partly recalled after delivery. Whole-participant nonresponse remains unresolved.
S2-55 | Main reference 54<br>The Efficacy of Transcutaneous Tibial Nerve Stimulation as an Adjunct to Standard Rehabilitation for Postpartum Pelvic Floor Dyssynergic Defecation in Primiparous Women: A Randomized Controlled Trial (2026)<br>Randomized trial<br>40 primiparas with postpartum dyssynergic defecation; 20 per group. Mean baseline 8.1	TTNS plus shared rehabilitation versus sham plus rehabilitation; constipation score, clinical response, PFM sEMG AND anorectal manometry.	Symptom, sEMG AND anorectal manometry results reported. Clinical response 18/20 versus 13/20 does not support the stated significance (two-sided Fisher p=0.127); no transit measurement.	Direct postpartum bowel/functional evidence<br>Core<br>No report family recorded	A | R<br>40 randomized with sham and assessor blinding; provider not blinded. Clinical-efficacy table reports significance inconsistent with 18/20 versus 13/20 (two-sided Fisher p=0.127). Measurement reporting needs verification.

versus 8.4 weeks postpartum; 6-week treatment.<br>https://doi.org/10.1007/s00192-026-06564-0				
S2-57 | Main reference 55<br>Effect of Holistic Postural Rehabilitation Combined With Electrical Stimulation and Biofeedback on Postpartum Pelvic Floor Dysfunction: A Multicenter Randomized Controlled Trial (2025)<br>Randomized trial<br>120 allocated (60/60); 87 analyzed (41/46) across three centres. Entry 42 days postpartum; assessments through 3 months. Results also describes 87 as enrolled.<br>https://doi.org/10.31083/CEOG44489	Postural rehabilitation plus electrical stimulation/biofeedback; PFDI-20 and UI	No between-group CRADI-8 benefit (3 months p=0.91). Within-group change does not establish bowel superiority of holistic rehabilitation over Kegels plus common stimulation/biofeedback.	Indirect mechanistic or intervention support<br>Supporting<br>No report family recorded	A | R<br>120 allocated (60/60), 87 analyzed (41/46), but Results describes 87 enrolled. Concealment/masked party unclear. Table denominators and baseline entries conflict; reported t/p values inconsistent; center clustering not addressed.

Abbreviations: ARM, anorectal manometry; IAP, intra-abdominal pressure; PFM, pelvic-floor muscle; sEMG, surface electromyography; PFME/PFMT, pelvic-floor muscle exercise/training; PFPT, pelvic-floor physical therapy; OASI, obstetric anal sphincter injury; DRA/IRD, diastasis recti/inter-recti distance; TTNS, transcutaneous tibial nerve stimulation; WBV, whole-body vibration; POP-Q, pelvic organ prolapse quantification; CRADI-8, colorectal–anal distress scale; CRAIQ-7, colorectal–anal impact scale�MAP and POPRACT denote report families, not separate outcomes�

Supplementary Table S3 Item-Level Methodological Appraisal Matrices for Included Primary Studies

Unreported safeguards are generally unclear (U); an explicitly absent safeguard is no (N)□Y denotes a met criterion; NA denotes a criterion not applicable to the actual study question□

R Randomized trial criteria

Q1 random allocation; Q2 allocation concealed; Q3 groups comparable at baseline; Q4 participants masked; Q5 providers masked; Q6 outcome assessors masked; Q7 same care except the assigned intervention; Q8 follow-up complete or losses appropriately analysed; Q9 analysis by assigned groups; Q10 outcomes assessed consistently; Q11 outcome measurement reliable; Q12 statistical approach appropriate; Q13 design and departures appropriately addressed.

Report	Q1	Q2	Q3	Q4	Q5	Q6	Q7	Q8	Q9	Q10	Q11	Q12	Q13
S2-18	Y	U	Y	N	N	N	Y	N	Y	Y	Y	U	Y
S2-37	Y	U	Y	N	N	U	Y	U	U	Y	Y	U	Y
S2-41	Y	U	Y	N	N	Y	Y	Y	Y	Y	U	U	Y
S2-55	Y	Y	Y	Y	N	Y	Y	Y	Y	Y	U	N	Y
S2-57	U	U	U	N	N	U	U	N	U	Y	U	N	U

C Cohort report criteria

Q1 groups from comparable populations; Q2 exposure assessed consistently; Q3 exposure assessment valid and reliable; Q4 confounders identified; Q5 confounding addressed; Q6 incident outcome absent at baseline when applicable; Q7 outcome assessment valid and reliable; Q8 follow-up long enough for the question; Q9 losses described and explored; Q10 missing follow-up addressed; Q11 statistical approach appropriate.

Report	Q1	Q2	Q3	Q4	Q5	Q6	Q7	Q8	Q9	Q10	Q11
S2-5	Y	Y	U	Y	N	N	U	Y	N	N	Y
S2-6	Y	Y	Y	Y	N	N	Y	Y	U	N	Y
S2-15	Y	Y	U	Y	N	N	Y	Y	Y	N	U
S2-21	Y	Y	Y	Y	Y	N	Y	Y	Y	N	Y
S2-23	Y	Y	Y	Y	Y	N	Y	Y	Y	N	Y

S2-24	Y	Y	Y	Y	N	Y	Y	Y	N	N	Y
S2-29	Y	Y	Y	Y	Y	N	Y	Y	Y	U	Y
S2-31	Y	Y	U	Y	Y	N	Y	Y	Y	N	Y
S2-38	Y	Y	Y	Y	Y	N	Y	Y	Y	U	Y
S2-39	N	Y	Y	Y	N	N	Y	Y	U	N	N
S2-42	Y	Y	Y	Y	Y	N	Y	Y	U	Y	Y
S2-48	NA	Y	Y	Y	N	N	Y	Y	N	N	U
S2-49	Y	Y	Y	Y	Y	N	Y	Y	Y	N	Y
S2-54	Y	Y	Y	Y	Y	N	Y	Y	U	N	Y

X Analytical cross-sectional report criteria

Q1 eligibility specified; Q2 participants and setting described; Q3 exposure assessment valid and reliable; Q4 objective criteria for the condition when applicable; Q5 confounders identified; Q6 confounding addressed; Q7 outcome assessment valid and reliable; Q8 statistical approach appropriate.

**Report**	**Q1**	**Q2**	**Q3**	**Q4**	**Q5**	**Q6**	**Q7**	**Q8**
S2-17	Y	Y	Y	Y	Y	N	Y	Y
S2-19	Y	Y	Y	Y	Y	N	Y	Y
S2-20	Y	Y	U	U	Y	U	U	U
S2-22	Y	Y	Y	Y	Y	Y	Y	Y
S2-32 complementary	Y	Y	U	Y	Y	N	Y	U
S2-35	Y	Y	Y	Y	Y	Y	U	Y
S2-36	Y	Y	Y	Y	Y	Y	Y	Y
S2-43	Y	Y	Y	Y	Y	Y	U	Y

Q Quasi-experimental report criteria

Q1 cause precedes the outcome; Q2 comparison participants comparable; Q3 other care comparable; Q4 control comparison present; Q5 repeated pre/post observations; Q6 follow-up complete or losses explored; Q7 outcome assessment consistent; Q8 outcome assessment reliable; Q9 statistical approach appropriate.

**Report**	**Q1**	**Q2**	**Q3**	**Q4**	**Q5**	**Q6**	**Q7**	**Q8**	**Q9**
S2-12	Y	Y	Y	N	N	U	Y	U	Y
S2-14	Y	N	U	Y	N	U	N	U	N
S2-30	Y	Y	U	N	N	N	Y	U	N

S2-40	Y	Y	U	N	N	N	Y	U	Y
S2-50	Y	Y	U	N	N	U	Y	U	U

K Case–control report criteria

Q1 cases and controls comparable; Q2 matching appropriate if used; Q3 case/control ascertainment consistent; Q4 exposure assessment valid and reliable; Q5 exposure assessment consistent; Q6 confounders identified; Q7 confounding addressed; Q8 outcome ascertainment valid and consistent; Q9 exposure window sufficient; Q10 statistical approach appropriate.

Report	Q1	Q2	Q3	Q4	Q5	Q6	Q7	Q8	Q9	Q10
S2-46	Y	NA	Y	Y	Y	Y	Y	Y	U	Y

P Prevalence report criteria

Q1 sampling frame appropriate; Q2 selection appropriate; Q3 sample sufficiently large; Q4 participants and setting described; Q5 analysis covers the sample; Q6 condition ascertainment valid; Q7 measurement reliable and consistent; Q8 statistical approach appropriate; Q9 response rate or nonresponse management adequate.

Report	Q1	Q2	Q3	Q4	Q5	Q6	Q7	Q8	Q9
S2-32	Y	U	Y	Y	U	Y	Y	U	U
S2-52	Y	U	U	Y	U	U	U	U	U
